# Publisher Correction: Legume rhizodeposition promotes nitrogen fixation by soil microbiota under crop diversification

**DOI:** 10.1038/s41467-024-47979-x

**Published:** 2024-04-26

**Authors:** Mengjie Qiao, Ruibo Sun, Zixuan Wang, Kenneth Dumack, Xingguang Xie, Chuanchao Dai, Ertao Wang, Jizhong Zhou, Bo Sun, Xinhua Peng, Michael Bonkowski, Yan Chen

**Affiliations:** 1 State Key Laboratory of Soil and Sustainable Agriculture, Institute of Soil Science, Chinese Academy of Sciences, Nanjing, 210008 China; 2 University of Chinese Academy of Sciences, Beijing, 100049 China; 3 Anhui Province Key Lab of Farmland Ecological Conservation and Nutrient Utilization, College of Resources and Environment, Anhui Agricultural University, Hefei, 230036 China; 4 College of Land Resource and Environment, Jiangxi Agricultural University, Nanchang, 330045 China; 5 Terrestrial Ecology, Institute of Zoology, University of Cologne, Zülpicher Str 47b, Cologne, 50674 Germany; 6 National Key Laboratory of Plant Molecular Genetics, Center for Excellence in Molecular Plant Sciences, Institute of Plant Physiology and Ecology, Chinese Academy of Sciences, Shanghai, 200032 China; 7 College of Life Sciences, Nanjing Normal University, Nanjing, 210023 China; 8 Institute for Environmental Genomics and Department of Microbiology and Plant Biology, University of Oklahoma, Norman, 73019 USA; 9 Cluster of Excellence on Plant Sciences (CEPLAS), University of Cologne, Cologne, 50674 Germany

**Keywords:** Agroecology, Microbial ecology, Metabolomics

Correction to: *Nature Communications* 10.1038/s41467-024-47159-x, published online 04 April 2024

In this article the affiliation ‘State Key Laboratory of Soil and Sustainable Agriculture, Institute of Soil Science, Chinese Academy of Sciences, Nanjing 210008, China’ for Xinhua Peng was missing. The original article has been corrected.

