# Supplementary material for: Legume rhizodeposition promotes nitrogen fixation by soil microbiota under crop diversification
Source: Nat Commun. 2024 Apr 4;15:2924. doi: 10.1038/s41467-024-47159-x (PMC10995168; doi:10.1038/s41467-024-47159-x)
Supplement: Supplementary file 1 — Supplementary Information [file 41467_2024_47159_MOESM1_ESM.pdf]

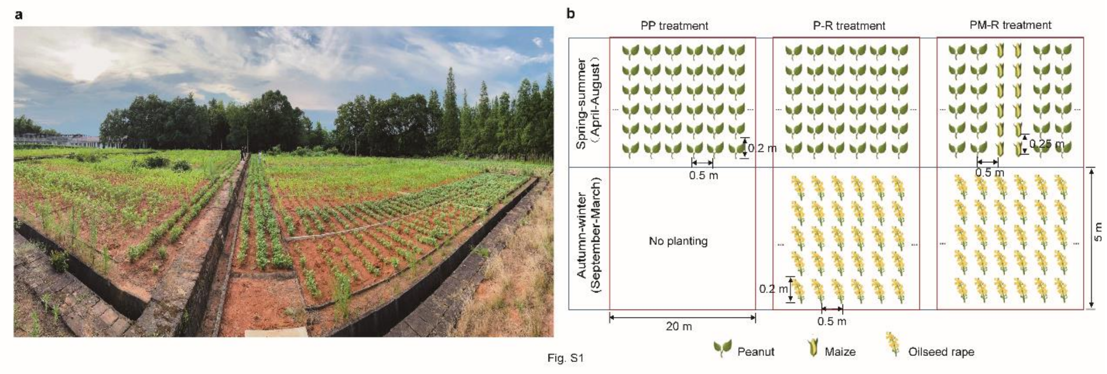

Fig. S1 Design of the crop diversification field experiment (from 2011-present). (**a**) Site photo of the long-term diversification experiment. (**b**) Diagram of diversified planting patterns. Experiment was begun in 2011 and located at the Red Soil Ecological Experimental Station of the Chinese Academy of Sciences in Jiangxi of China. PP, P-R and PM-R represent peanut monocropping, peanut-oilseed rape rotation, and peanut-maize intercropping rotated with oilseed rape, respectively.

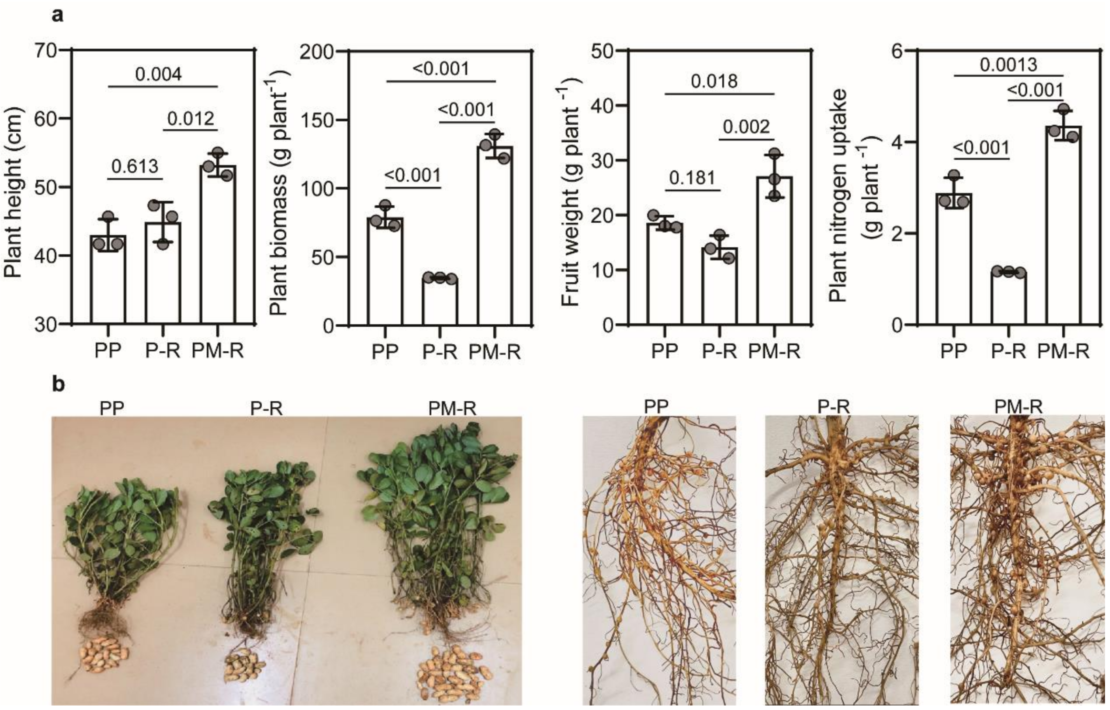

Fig. S2 Effect of crop diversification on peanut performance. (**a**) Effect on peanut plant height, biomass and fruit weight and N uptake. The error bars with p values between groups were calculated using one-way ANOVA and Tukey's post-hoc tests (two-sided, n=3 biologically independent replicates from 9 samples per treatment) (**b**) Photos of peanut growth and root nodulation. PP, P-R and PM-R represent peanut monocropping, peanut-oilseed rape rotation, and peanut-maize intercropping rotated with oilseed rape, respectively.

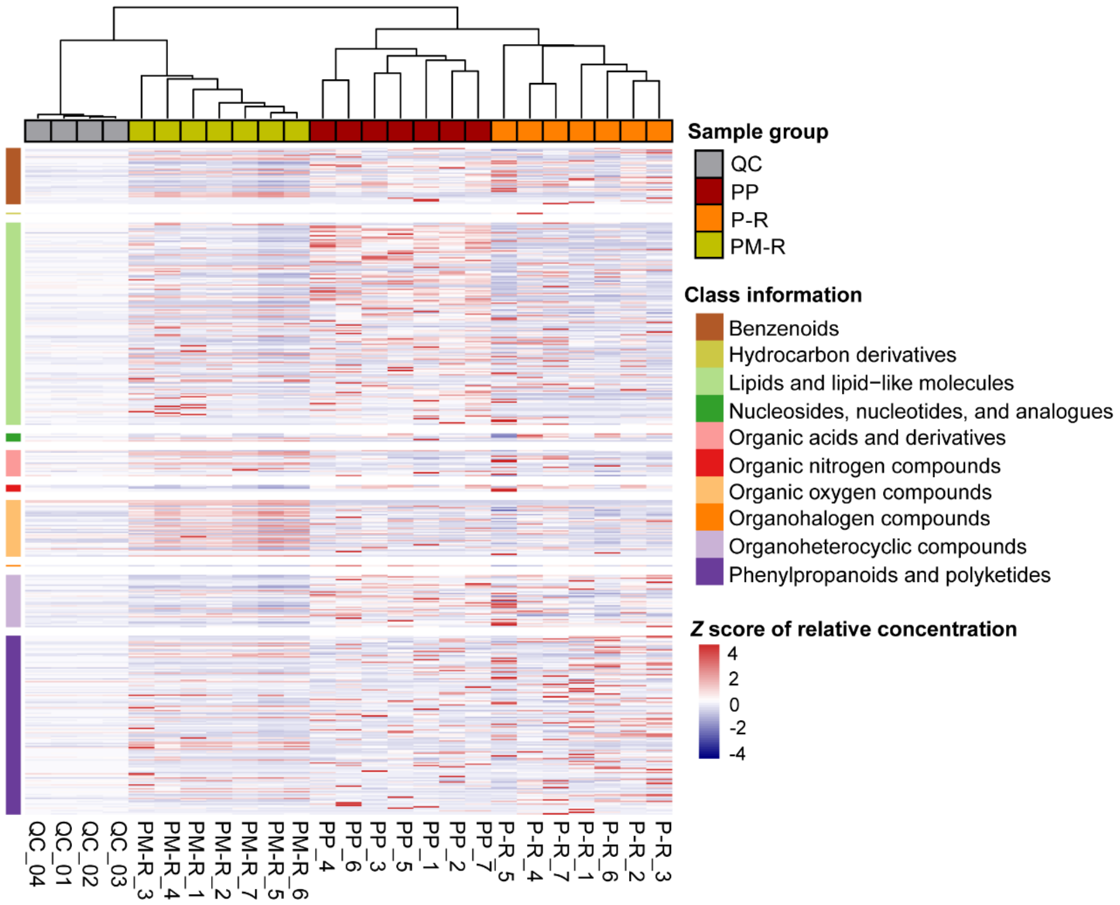

Fig. S3 Heatmap and hierarchical clustering of peanut rhizosphere metabolites in the different cropping systems based on pairwise Euclidean distance. QC, quality control samples (composed of a small aliquot of each sample); PP, P-R and PM-R represent peanut monocropping, peanut-oilseed rape rotation, and peanut-maize intercropping rotated with oilseed rape, respectively.

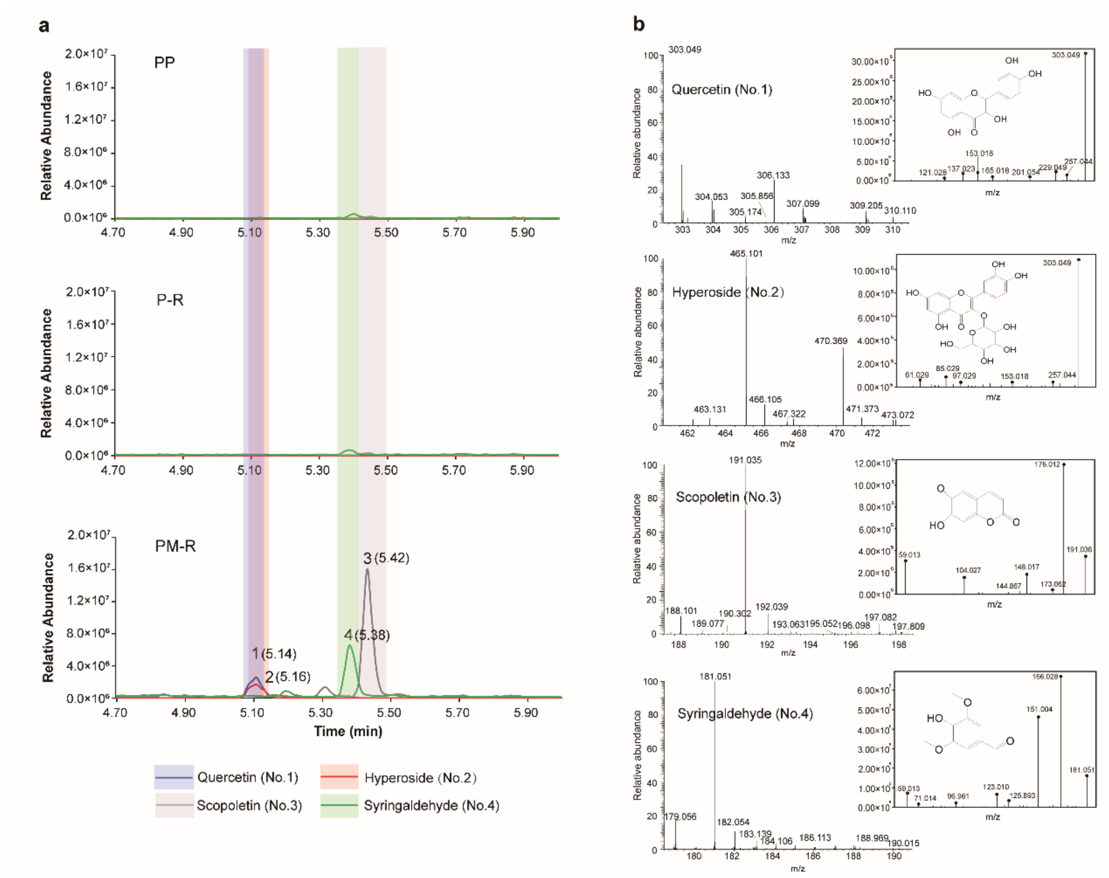

Fig. S4 Detection of four typical enriched metabolites in nontargeted metabolic profiling using ultra-performance liquid chromatography-tandem mass spectrometry (UHPLC–MS/MS). (**a**) Extracted ion chromatogram (EIC) of different metabolites from the soil metabolic profiling; (**b**) Primary and corresponding secondary mass spectrum of each typical metabolite. PP, P-R and PM-R represent peanut monocropping, peanut-oilseed rape rotation, and peanut-maize intercropping rotated with oilseed rape, respectively.

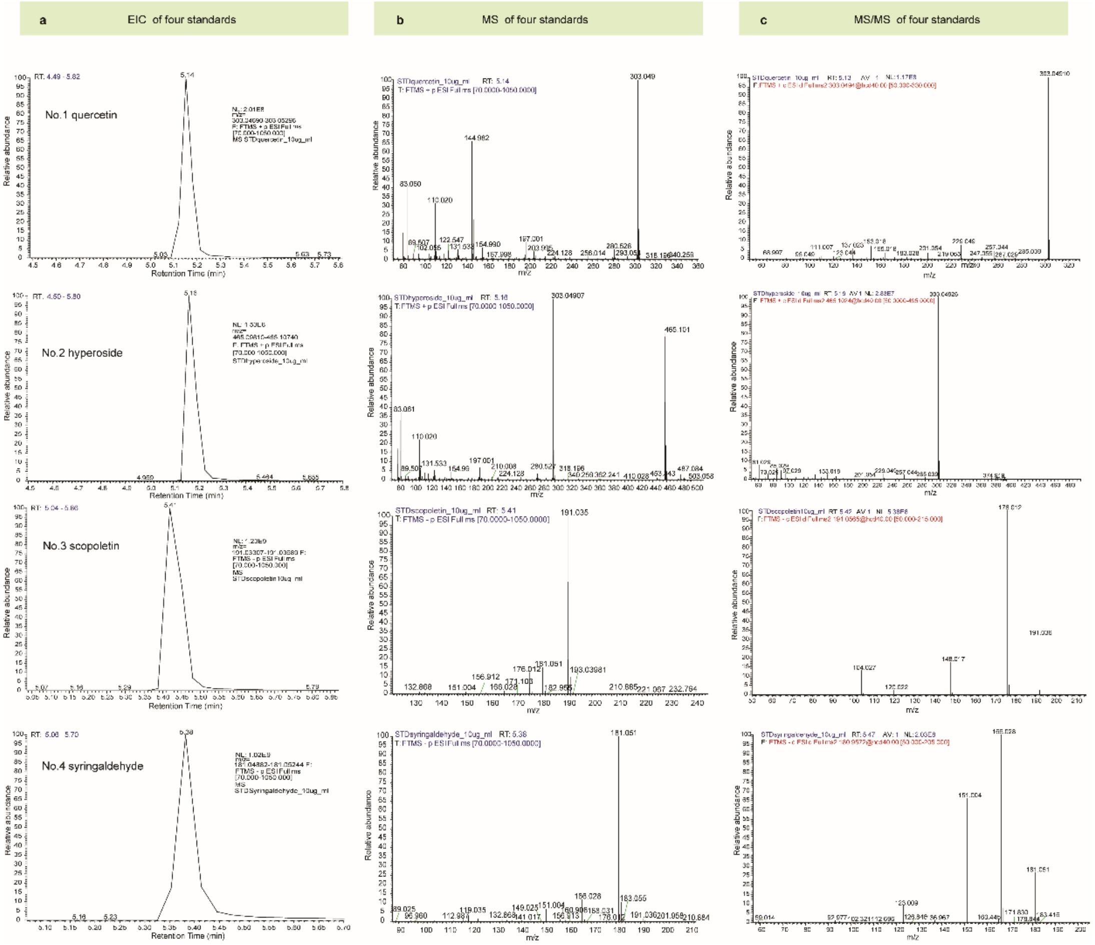

Fig. S5 Mass spectra of four metabolite standards (quercetin, hyperoside, scopoletin and syringaldehyde) using UHPLC–MS/MS. (**a**) Extracted ion chromatogram (EIC) of quercetin, hyperoside, scopoletin and syringaldehyde standards; (**b**) Primary mass spectrum (MS) of the four standards. (**c**) Secondary mass spectrum (MS/MS) of the four standards.

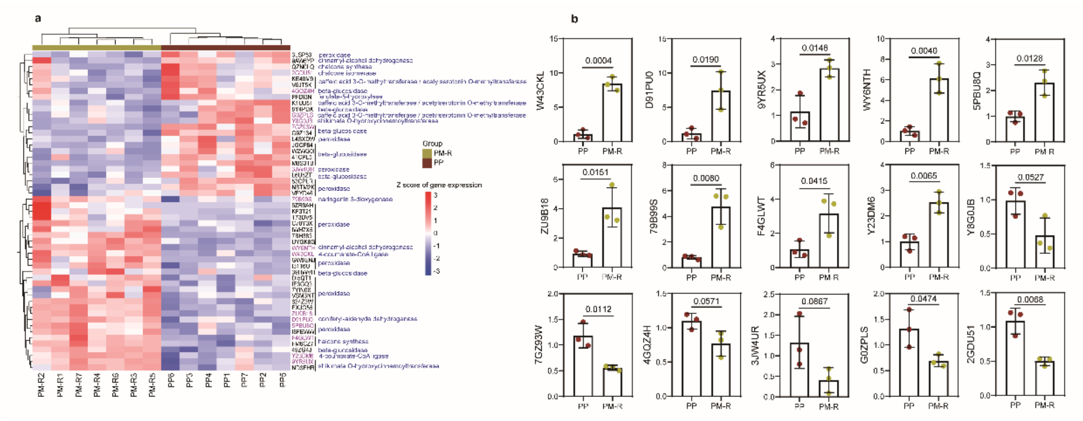

Fig. S6 Differentially expressed gene (DEG) profile of peanut root transcripts and validation of representative gene expressions. (**a**) DEG profile of transcripts associated with phenylpropanoid and flavonoid biosynthesis (KO00940; KO00941). (**b**) Validation of representative up-and down-regulated gene expressions by real-time polymerase chain reaction. Gene IDs marked with pink in A were selected for validation. Data are shown as $2^{-\Delta\Delta Ct}$ and presented as the mean ± SD (n=3 biologically independent replicates from 9 independent samples per treatment). The error bars with p values between groups were calculated using by two-sided t-test. PP, peanut roots from peanut monocropping; PM-R, peanut roots from peanut maize intercropping rotated with oilseed rape.

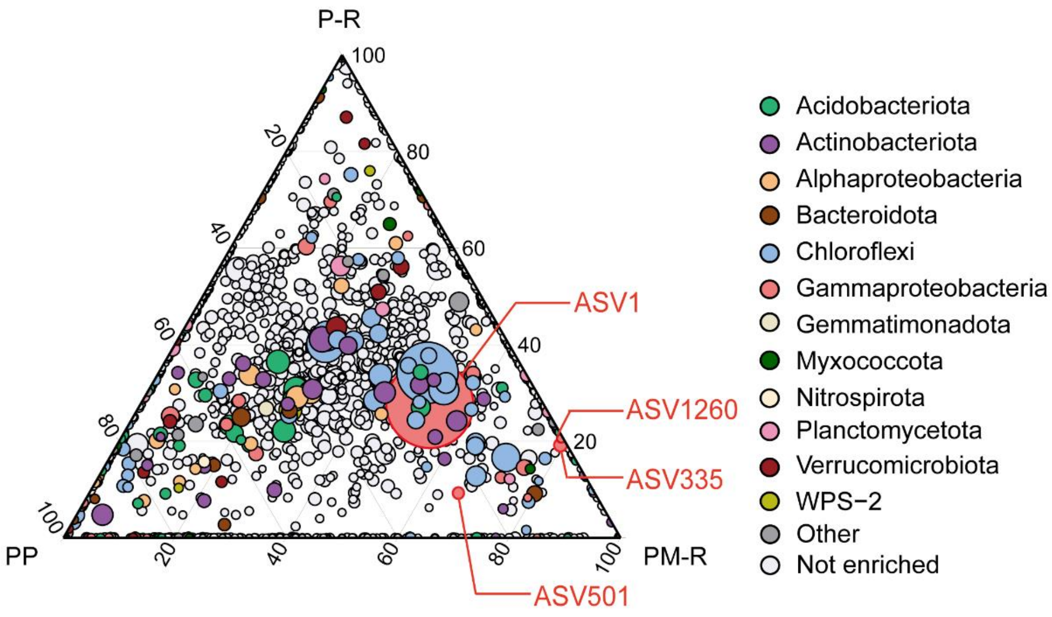

Fig. S7 Ternary plot of the shared bacterial communities among the different peanut rhizosphere soils. The grey circles represent ASVs whose relative abundances were not significantly different among the samples. The circles with other colours represent the phyla of the enriched ASVs, as shown in Fig. 3D. The circle size represents the relative abundance of the corresponding bacterial ASV. PP, P-R and PM-R represent peanut monocropping, peanut-oilseed rape rotation, and peanut-maize intercropping rotated with oilseed rape, respectively.

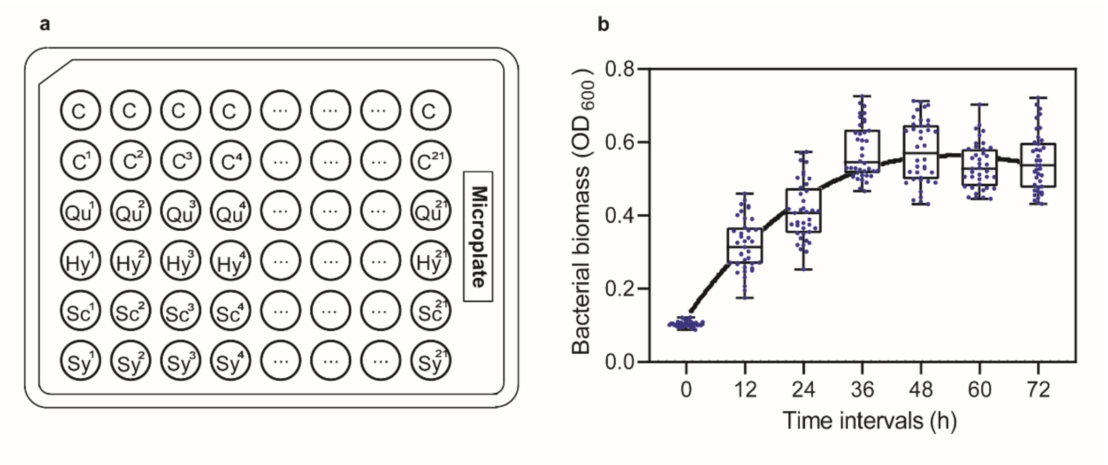

Fig. S8 Bacterial microplate cultures and growth curve. (**a**) Diagram of bacterial micro-plate (96 wells) cultures with and without metabolite addition. The number in the upper right of the well represents the number of the cultured bacterial strain (listed in Table S8). Each sample was conducted with 6 replicates. C, 1/5 TSB medium; Qu, 1/5 TSB medium with quercetin addition; Hy, 1/5 TSB medium with hyperoside addition; Sc, 1/5 TSB medium with Scopoletin addition; Sy, 1/5 TSB medium with syringaldehyde addition. (**b**) The growths of selected bacterial isolates in 1/5 TSB medium were measured for 72 hours (n=40 biological independent samples). Box plots indicate median (black line), 25th, 75th percentile (box) and 5th and 95th percentile (whiskers). Based on microbial growth curve (solid black line), we determined that bacteria were in the logarithmic growth phase during 12st-48th hour.

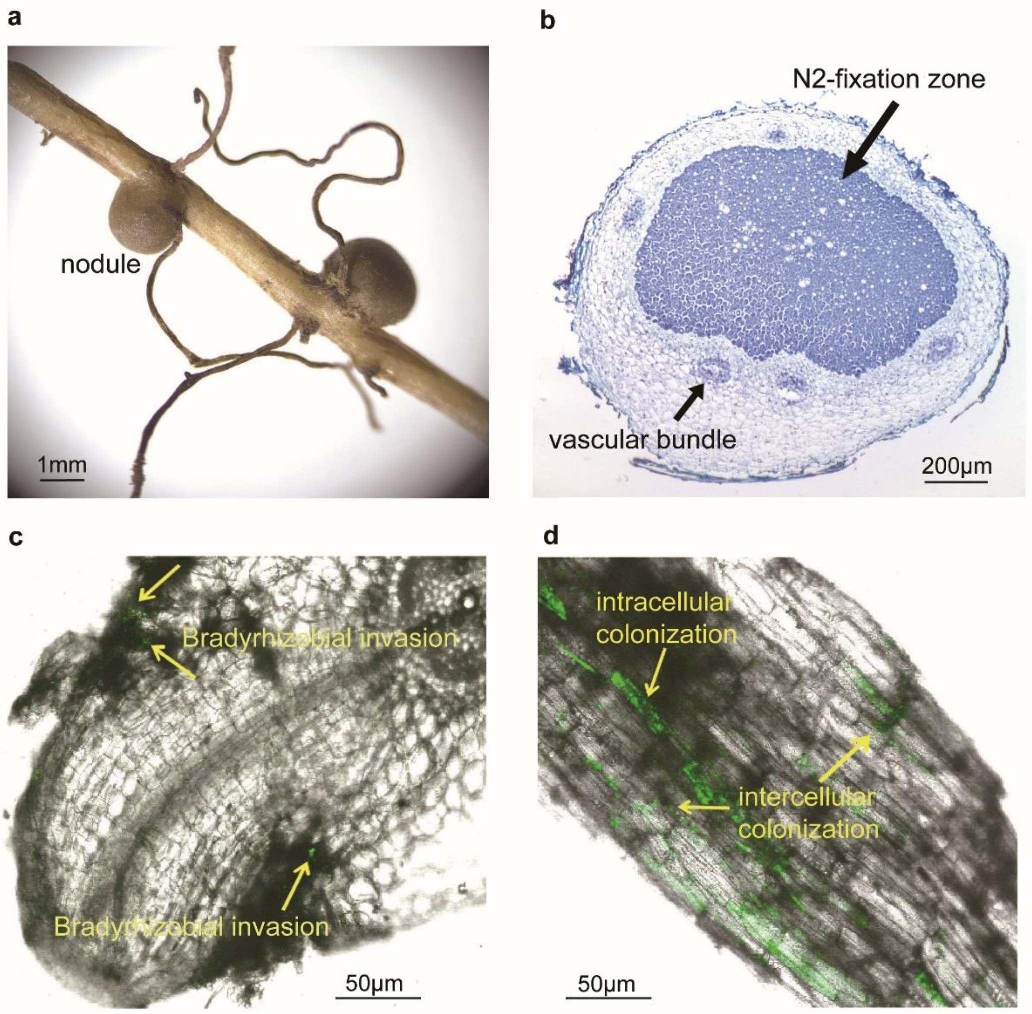

Fig. S9 The root colonization and nodulation of *Bradyrhizobium* N47 in host plant. (**a**) Nodules of peanut root after *Bradyrhizobium* N47 inoculation for 30 days using stereoscopic microscope. Scale bar, 1 mm. (**b**) Representative toluidine blue-stained section of nodule structure of 20 nodule sections. Scale bar, 200 μm.(**c-d**) Lateral root invasion and colonization by *Bradyrhizobium* N47. The strain was tagged by green fluorescent protein (GFP). The fluorescent signal of GFP was detected by fluorescence microscope. Scale bar, 50 μm.

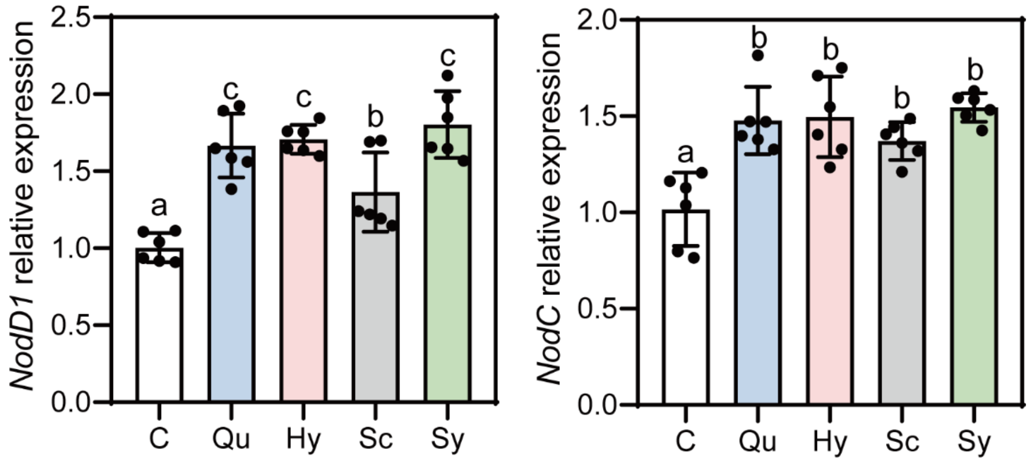

Fig. S10 Expression of *Sinorhizobium meliloti* (strain1021) *Nod* genes, measured 10h after addition of metabolites. The error bars with lowercase represent significant differences between groups ($p<0.05$) via one-way ANOVA and Tukey's post-hoc tests (two-sided, n=6 biologically independent samples per treatment). C, control; Br, *Bradyrhizobium* N47; Qu, quercetin; Hy, hyperoside; Sc, scopoletin; Sy, syringaldehyde.

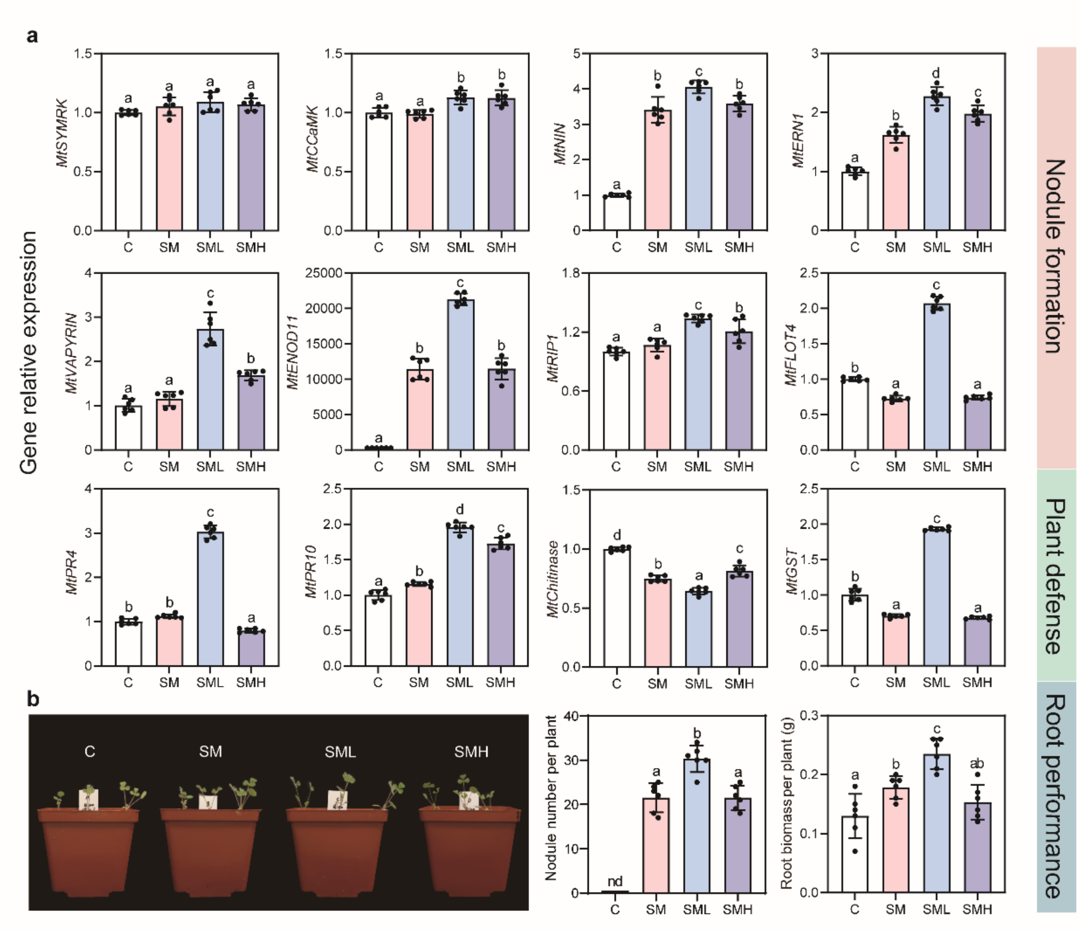

Fig. S11 Effect of coumarin scopoletin on the root gene expression of *Medicago truncatula.* (**a**) Genes involved in root nodule formation and host defense. (**b**) Photograph of plant growth on the 30th day. The data are shown as the mean ± SD. The error bars with lowercase indicate significant differences between groups ($p<0.05$) via one-way ANOVA and Tukey's post-hoc tests (two-sided, n=6 biologically independent samples). C, control; SM, seedling inoculated with rhizobia *Sinorhizobium meliloti*; SML, seedling inoculated with rhizobia *S. meliloti* strain1021 and low concentration (5 μg mL^{-1}) of scopoletin; SMH, seedling inoculated with rhizobia *S. meliloti* strain1021 and high concentration (50 μg mL^{-1}) of scopoletin.
