# Supplementary material for: Legume rhizodeposition promotes nitrogen fixation by soil microbiota under crop diversification
Source: Nat Commun. 2024 Apr 4;15:2924. doi: 10.1038/s41467-024-47159-x (PMC10995168; doi:10.1038/s41467-024-47159-x)
Supplement: Supplementary file 14 — Reporting Summary [file 41467_2024_47159_MOESM14_ESM.pdf]

nature portfolio

Corresponding author(s): Yan Chen

Last updated by author(s): Mar 10, 2024

Reporting Summary

Nature Portfolio wishes to improve the reproducibility of the work that we publish. This form provides structure for consistency and transparency in reporting. For further information on Nature Portfolio policies, see our Editorial Policies and the Editorial Policy Checklist.

Statistics

For all statistical analyses, confirm that the following items are present in the figure legend, table legend, main text, or Methods section.

n/a	Confirmed	
☐	☒	The exact sample size (n) for each experimental group/condition, given as a discrete number and unit of measurement
☐	☒	A statement on whether measurements were taken from distinct samples or whether the same sample was measured repeatedly
☐	☒	The statistical test(s) used AND whether they are one- or two-sided<br>*Only common tests should be described solely by name; describe more complex techniques in the Methods section.*
☐	☒	A description of all covariates tested
☐	☒	A description of any assumptions or corrections, such as tests of normality and adjustment for multiple comparisons
☐	☒	A full description of the statistical parameters including central tendency (e.g. means) or other basic estimates (e.g. regression coefficient) AND variation (e.g. standard deviation) or associated estimates of uncertainty (e.g. confidence intervals)
☐	☒	For null hypothesis testing, the test statistic (e.g. F, t, r) with confidence intervals, effect sizes, degrees of freedom and P value noted<br>*Give P values as exact values whenever suitable.*
☒	☐	For Bayesian analysis, information on the choice of priors and Markov chain Monte Carlo settings
☐	☒	For hierarchical and complex designs, identification of the appropriate level for tests and full reporting of outcomes
☐	☒	Estimates of effect sizes (e.g. Cohen's d, Pearson's r), indicating how they were calculated

Our web collection on statistics for biologists contains articles on many of the points above.

Software and code

Policy information about availability of computer code

Data collection	We didn't use any commercial, open source and custom code to collect the data in this study.
Data analysis	The data (over three treatment groups, follows a normal distribution) of field physiological traits, soil physicochemical properties, microbial alpha-diversity and qPCR of genes involved in rhizobial colonization, plant defense and nodulation in peanut and Medicago that were analysed using Tukey's post-hoc tests for multiple comparisons to explore the significance of the difference between pairs of the treatments when One-way analysis of variance (ANOVA) terms were significant. The data of (two treatment groups, follows a normal distribution) root transcriptome and gene expressions between PP and PM-R two treatment groups, enrichment of the four typical metabolites (quercetin, hyperoside, scopoletin, and syringaldehyde), isolates' growth rates were analysed using two-tailed unpaired t-tests according to F-test results to compare significant differences. The least squares regression was performed to detect the relationships between root nodulation, soil 15N2 fixation, plant biomass and rhizosphere nitrogen (including total, ammonia and nitrate nitrogen). The assumptions of homoscedasticity and normality of the residuals of the regression models were checked to confirm the reliability of the models. The hierarchical clustering based on Euclidean distance and heatmaps of metabolites and transcriptional functional genes were performed with the "heatmap" package. Principal Component Analysis (PCA) and similarity analysis (ANOSIM) of metabolome and transcriptome data based on the pairwise Euclidean distance was performed to reveal the dissimilarities among different cropping treatment groups using the "stats" package. Multivariate homogeneity of groups dispersions of bacterial amplicon sequencing data based on Bray–Curtis distance was conducted using "vegan" package. Ternary plots for identifying specific enriched microbial taxa in the cropping treatment groups were generated with the "vcd" package.<br>The authors declare that the R (R 4.3.1) codes used to generate the results reported in this study are available in this paper. The R code supporting the findings presented here is available from the GitHub Repository (https://github.com/Pop-rainbow/paper/blob/main/NCOMMS%2023-15056%20Code%20file.docx).

For manuscripts utilizing custom algorithms or software that are central to the research but not yet described in published literature, software must be made available to editors and reviewers. We strongly encourage code deposition in a community repository (e.g. GitHub). See the Nature Portfolio guidelines for submitting code & software for further information.

Data

Policy information about availability of data

All manuscripts must include a data availability statement. This statement should provide the following information, where applicable:

- Accession codes, unique identifiers, or web links for publicly available datasets
- A description of any restrictions on data availability
- For clinical datasets or third party data, please ensure that the statement adheres to our policy

The 16S rRNA gene sequences and peanut transcriptome sequences were deposited into the Sequence Read Archive (RDA) of the National Centre for Biotechnology Information (NCBI) database under the accession numbers PRJNA718421 and PRJNA790729. The sequences of all isolates (including deleted duplicate data) were deposited into the National Centre for Biotechnology Information (NCBI) database under accession number MW856489-MW856613. Raw nontarget metabolite data were deposited into the European Molecular Biology Laboratory (EMBL-EBI) MetaboLights database under accession number MTBLS6537. Source data are provided with this paper.

Research involving human participants, their data, or biological material

Policy information about studies with human participants or human data. See also policy information about sex, gender (identity/presentation), and sexual orientation and race, ethnicity and racism.

Reporting on sex and gender	We didn't collect the terms sex and gender in this study.
Reporting on race, ethnicity, or other socially relevant groupings	We didn't mention any covariate-relevant population characteristics of the human research participants in this study.
Population characteristics	We didn't mention any covariate-relevant population characteristics of the human research participants in this st
Recruitment	No participant was recruited.
Ethics oversight	No organization was mentioned in this study.

Note that full information on the approval of the study protocol must also be provided in the manuscript.

Field-specific reporting

Please select the one below that is the best fit for your research. If you are not sure, read the appropriate sections before making your selection.

☒ Life sciences ☐ Behavioural & social sciences ☐ Ecological, evolutionary & environmental sciences

For a reference copy of the document with all sections, see nature.com/documents/nr-reporting-summary-flat.pdf

Life sciences study design

All studies must disclose on these points even when the disclosure is negative.

Sample size	For the long-term field experiment, 48 plant samples were collected for physiological and transcription analysis (three treatments with nine replicates of plant samples for each were collected for plant physiological determination; seven replicates of plant samples for each were collected for root transcriptomic analysis); 102 soil samples were collected for chemical, metabolic and high-throughput sequencing analysis (nine replicates of rhizosphere/bulk soil samples for each were collected for soil chemical property analysis; nine replicates of rhizosphere soil for each were collected for 16s high-throughput sequencing; seven replicates of rhizosphere soil for each were collected for metabolic analysis). For isolated bacterial cultivation, total 810 wells ((4 metabolites+control) × 27 isolates x 6 replicates) were cultivated. For specific N2-fixing isolate and plant interaction, 150 samples were conducted for gene expressions (5 treatments × 5 genes × six replicates).
Data exclusions	For metabolic analysis of field soil samples, due to the two samples per treatment were set aside for pre-analysis but were subsequently lost due to a broken freezer, hence for the metabolite analysis n=7 instead of n=9.
Replication	For the long-term field experiment, three treatments with nine replicates of plant samples for each were collected for plant physiological determination; seven replicates of plant samples for each were collected for root transcriptomic analysis;nine replicates of rhizosphere/bulk soil samples for each were collected for soil chemical property analysis; nine replicates of rhizosphere soil for each were collected for 16s high-throughput sequencing; seven replicates of rhizosphere soil for each were collected for metabolic analysis. For 27 isolated bacterial cultivation, total 810 wells ((4 metabolites+control) × 27 isolates x 6 replicates) were cultivated. For specific N2-fixing isolate and plant interaction, each samples were conducted with six replicates.
Randomization	All the field plants and corresponding in each treatment were selected randomly. In the field experiment, we have three treatments (PP, P-R and PM-R). Each treatment is conducted with three plots (3 replicates). The size of each plot is 100 m2 (20m×5m) . We planned to randomly collect 3 soil replicates in each plot. Therefore, we would have 9 replicates for each treatment. Considering the large area of each plot but the small area of soil sampling (bulk soil 15cm×15cm, rhizosphere soil from three plants) would affect the uniformity of these representative samples, we improved our methods for sample collection: we divided the 100m2 area into 3 subplots and collected 6 soil samples in an S-shape in each subplot. Then these 6 samples were mixed into a composite sample. Therefore, three composite samples were collected from each plot, and nine were collected from each treatment. Compared with collected samples, our collections cover the entire area of plots and

	minimize soil heterogeneity due to sampling distance. During our statistical analysis of field experiment, we calculated the mean of all samples (n=3) for each plot to ensure a random selection for ANOVA analysis. Therefore, the replicates for each field treatment were three instead of nine.
Blinding	Blind was not possible. Our subjects are not humans.

Reporting for specific materials, systems and methods

We require information from authors about some types of materials, experimental systems and methods used in many studies. Here, indicate whether each material, system or method listed is relevant to your study. If you are not sure if a list item applies to your research, read the appropriate section before selecting a response.

Materials & experimental systems

n/a	Involved in the study
☒	☐ Antibodies
☒	☐ Eukaryotic cell lines
☒	☐ Palaeontology and archaeology
☒	☐ Animals and other organisms
☒	☐ Clinical data
☒	☐ Dual use research of concern
☐	☒ Plants

Methods

n/a	Involved in the study
☒	☐ ChIP-seq
☒	☐ Flow cytometry
☒	☐ MRI-based neuroimaging

Dual use research of concern

Policy information about dual use research of concern

Hazards

Could the accidental, deliberate or reckless misuse of agents or technologies generated in the work, or the application of information presented in the manuscript, pose a threat to:

No	Yes
☒	☐ Public health
☒	☐ National security
☒	☐ Crops and/or livestock
☒	☐ Ecosystems
☒	☐ Any other significant area

Experiments of concern

Does the work involve any of these experiments of concern:

No	Yes
☒	☐ Demonstrate how to render a vaccine ineffective
☒	☐ Confer resistance to therapeutically useful antibiotics or antiviral agents
☒	☐ Enhance the virulence of a pathogen or render a nonpathogen virulent
☒	☐ Increase transmissibility of a pathogen
☒	☐ Alter the host range of a pathogen
☒	☐ Enable evasion of diagnostic/detection modalities
☒	☐ Enable the weaponization of a biological agent or toxin
☒	☐ Any other potentially harmful combination of experiments and agents

Plants

Seed stocks	The seeds of peanut, maize and oilseed rape were purchased from Jiangsu Academy of Agricultural Science. The wild seeds of Medicago were given as a gift by Prof. Ertao Wang's group from Institute of Plant Physiology and Ecology, Chinese Academy of Sciences .
Novel plant genotypes	No novel plant genotypes is used in this study.
Authentication	No authentication in this study.
