# Supplementary material for: Legume rhizodeposition promotes nitrogen fixation by soil microbiota under crop diversification
Source: Nat Commun. 2024 Apr 4;15:2924. doi: 10.1038/s41467-024-47159-x (PMC10995168; doi:10.1038/s41467-024-47159-x)
Supplement: Supplementary file 16 — Description of Additional Supplementary Files [file 41467_2024_47159_MOESM16_ESM.pdf]

Supplementary Data 1
Effect of crop diversification on chemical properties in the peanut rhizosphere
Supplementary Data 2
Metabolites significantly enriched or depleted between treatments
Supplementary Data 3
Enrichment of phenylpropanoid and flavonoid biosynthesis pathways of KEGG (PM-R vs PP)
Supplementary Data 4
Functions of enriched genes in phenylpropanoid and flavonoid biosynthesis pathways of KEGG (PM-R vs PP)
Supplementary Data 5
Functional annotation of enriched genes involved in phenylpropanoid and flavonoid biosynthesis pathways based on Gene Ontology (GO)
Supplementary Data 6
Differences in the relative abundances of dominant phyla in the peanut rhizosphere
Supplementary Data 7
Information of enriched ASVs in the peanut rhizosphere of different cropping systems
Supplementary Data 8
Phylogenetic information of selected isolates for microplate inoculation
Supplementary Data 9
Information on four enriched ASVs in the most diverse cropping system and specific bacterial isolates
Supplementary Data 10
Primers for qRT-PCR for peanut symbiotic nodulation genes and phenylpropanoid biosynthesis genes
Supplementary Data 11
Primers for qRT-PCR for Medicago symbiotic nodulation and cytodefense genes
Supplementary Data 12
P values of figure 5b-d
